# Chitosan Effects on Plant Systems

**DOI:** 10.3390/ijms17070996

**Published:** 2016-06-23

**Authors:** Massimo Malerba, Raffaella Cerana

**Affiliations:** 1 Dipartimento di Biotecnologie e Bioscienze, Università degli Studi di Milano-Bicocca, Piazza della Scienza 2, 20126 Milano, Italy; massimo.malerba@unimib.it; 2 Dipartimento di Scienze dell’Ambiente e del Territorio e di Scienze della Terra, Università degli Studi di Milano-Bicocca, Piazza della Scienza 1, 20126 Milano, Italy

**Keywords:** chitosan, defense responses, fertilizers, herbicides, micronutrients, nanoparticles, pesticides

## Abstract

Chitosan (CHT) is a natural, safe, and cheap product of chitin deacetylation, widely used by several industries because of its interesting features. The availability of industrial quantities of CHT in the late 1980s enabled it to be tested in agriculture. CHT has been proven to stimulate plant growth, to protect the safety of edible products, and to induce abiotic and biotic stress tolerance in various horticultural commodities. The stimulating effect of different enzyme activities to detoxify reactive oxygen species suggests the involvement of hydrogen peroxide and nitric oxide in CHT signaling. CHT could also interact with chromatin and directly affect gene expression. Recent innovative uses of CHT include synthesis of CHT nanoparticles as a valuable delivery system for fertilizers, herbicides, pesticides, and micronutrients for crop growth promotion by a balanced and sustained nutrition. In addition, CHT nanoparticles can safely deliver genetic material for plant transformation. This review presents an overview on the status of the use of CHT in plant systems. Attention was given to the research that suggested the use of CHT for sustainable crop productivity.

## 1. Introduction

Chitosan is a natural, safe, and cheap biopolymer produced from chitin, the major constituent of arthropods exoskeleton and fungi cell walls and the second renewable carbon source after lignocellulosic biomass [1]. For industrial production, solid chitin is soaked in 40%–50% (*w*/*v*) NaOH. This process removes more than 80% of the acetyl residues and converts *N*-acetyl-d-glucosamine into β-1,4-d-glucosamine. The CHT preparations are heterogeneous as for deacetylation degree, molecular mass, polymerization degree, viscosity, acid dissociation constant (pKa value), and the term “chitosan” does not describe a unique compound, but a group of commercially available copolymers. This heterogeneity can greatly affect the physical properties of CHT, thus governing its biological applications [1].

In addition to its low-cost of production, CHT also possesses several favorable biological properties such as biodegradability, biocompatibility and non-allergenicity. CHT is susceptible to degradation by specific and nonspecific enzymes, and it shows low toxicity to humans [2]. All these characteristics make CHT very useful for several industries, namely cosmetology, food, biotechnology, pharmacology, and medicine [3,4].

The availability of industrial quantities of CHT in the 1980s enabled it to be tested as an agricultural tool. The results indubitably show that CHT can control many pre- and postharvest diseases on different crops. CHT treatment makes plants more tolerant to a wide range of soil and foliar pathogens and induces root nodulation [5], thus proposing CHT as a useful tool for agricultural sustainability. These published experimental data have been summarized in recent comprehensive reviews [6,7]. The huge value of these results motivated worldwide researchers to find more applications for CHT and its derivatives.

This paper reviews the current and ongoing research in these areas and it is organized into three sections: (1) physiological responses to CHT in plants; (2) application of CHT on crop/food plants; (3) use of CHT nanoparticles in agriculture as a delivery system.

## 2. Physiological Responses to CHT in Plants

CHT has been demonstrated to be a natural molecule that induces numerous biological responses in plants, dependent on its structure and concentration and on species and developmental stage of the plant. After the first report on the eliciting action of CHT in pea (*Pisum sativum* L.) and tomato (*Solanum lycopersicum* L.) plants [8], CHT was shown to enhance defense responses to abiotic and biotic stresses. An initial oxidative burst with hydrogen peroxide (H_2_O_2_) accumulation was observed in different plants supplied with CHT [6] as well as in plant cell cultures [9,10]. It is thought that this can lead to the induction of plant defense enzymes, and to the synthesis of secondary metabolites, such as polyphenolics, lignin, flavonoids, and phytoalexins observed in many plant species treated with CHT. These species include but are not limited to sunflower (*Helianthus annuus* L.) [11], papaya (*Carica papaya* L.) [12], litchi (*Litchi chinensis*) [13], grape (*Vitis vinifera* L.) [14], apricot (*Prunus armeniaca* L.) [15], loquat (*Eriobotrya japonica*) [16], soybean (*Glycine max* L.) [17], tomato (*Solanum lycopersicum* L.) [18], Greek oregano (*Origanum vulgare* ssp. hirtum) [19], butter lettuce (*Lactuca sativa* L.) [20], sweet basil (*Ocimum basilicum* L.) [21], and spinach (*Spinacia oleracea*) [22]. Other biochemical and molecular changes observed in plants fed with CHT include: callose apposition [23,24], increases in cytosolic Ca^2+^ [25], activation of MAP-kinases [26], plasma membrane H^+^-ATPase inhibition [27], chromatin alterations [28,29], synthesis of alkaloids [30], and phytoregulators (jasmonic acid, JA, and abscisic acid, ABA) [31,32].

### 2.1. Mechanism of CHT Action

The mode of action of CHT is not yet completely unraveled. The different mechanisms for its antimicrobial effect, summarized in Table 1, include interaction with plasma membrane phospholipids and histone proteins as well as chelation of trace metal elements [33,34,35,36,37,38,39,40,41].

For induction of defense responses in plants, CHT may directly affect gene expression interacting with chromatin [42] and/or it may bind to specific receptors. A CHT-binding protein belonging to the glycoprotein family of lectines has been isolated from leaves of *Brassica campestris* L. (ssp. *chinensis* (L.) Makino) [43]. The presence of CHT receptors is also suggested by the rapid activation of plasma membrane H^+^-ATPase in isolated vesicles from pulvini of *Mimosa pudica* and *Cassia fasciculata* [27]. In addition, CHT can induce a receptor-like kinase gene and a MAP kinase pathway, and the lysin motif receptor-like kinase chitin elicitor receptor kinase 1 (CERK1) seems to bind chitin and CHT as suggested by experiments with *A. thaliana* knockout mutants [44]. Interestingly, the lysin motif domains of CERK1 are also present in different legume receptors of Nod factors, chitin-related molecules produced by nitrogen-fixing bacteria [45]. On the other hand, in *Arabidopsis* seedlings Povero et al. [46] reported that the signaling by CHT does not use CERK1 and is perceived through a CERK1-independent pathway. Thus, the categorization of CERK1 as a CHT receptor is still uncertain and CHT remains “a Pathogen-Associated Molecular Pattern (PAMP) in search of a Pattern Recognition Receptor (PRR)” [29].

### 2.2. Signals Inside the Cell

After the recognition of the CHT molecule by a specific cellular receptor, second messenger(s) must transduce the signal to induce the physiological responses. Several papers report the involvement of molecules like reactive oxygen species (ROS), Ca^2+^, nitric oxide (NO), phytohormones in the CHT-mediated signaling pathway. As reported previously in this paper ROS, in particular H_2_O_2_ regulates several responses induced by CHT in many plant species [11,12,13,14,15,16,17,18,19,20,21,22]. Ca^2+^ regulates callose synthase activity in response to CHT elicitation in both monocotyledonous and dicotyledonous species [23,47], mediates the programmed cell death induced by CHT in soybean cells [25] and the cell death kinetic induced by CHT was delayed by treatment with a calcium channel blocker during tobacco necrosis virus infection of tobacco plants [48]. The possible signaling role of NO has been investigated in pearl millet seedlings where the degree of protection by CHT against pathogens was decreased by treatment with the NO synthase inhibitor LNAME (*N*-nitro-l-arginine methyl ester hydrochloride) or with the NO scavenger c-PTIO [2-(4-carboxyphenyl)-4,4,5,5-tetramethylimidazoline-1-oxyl-3-oxide potassium salt] [49] and in tomato cell cultures, where c-PTIO applied together with CHT decreased the level of phosphatidic acid (PA) production after pathogen attacks [50]. In tobacco leaves, c-PTIO and LNAME partly blocked the activation of Ser/Thr protein kinases and the activities of many defense-related enzymes induced by a chitooligosaccharide while the NO donor sodium nitroprusside induced the activation of kinases and enhanced the defense systems [51]. Recently, Malerba and Cerana obtained similar results in sycamore (*Acer pseudoplatanus* L.) cultured cells where c-PTIO prevented several stress responses induced by CHT [52]. As far as phytohormones are concerned, CHT induced a fast accumulation of JA in rice [53] and *Phaseolus vulgaris* [29]. A cDNA microarray/semiquantitative RT-PCR analyses of *Brassica napus* gene expression changes induced by CHT showed that CHT activated the plant self-defense through JA/ethylene signaling pathway [54]. In different plant/pathogen interactions, the degree and kinetics of callose synthesis are regulated by ABA [55]. Interestingly, the ABA inhibitor nordihydroguaiaretic acid, applied before CHT, decreased both callose synthesis and plant resistance to tobacco necrosis virus thus indicating the involvement of ABA in the CHT action [29]. Finally, the phytohormones indole-3-acetic acid and kinetin stimulated growth and CHT production of the fungus *Mucor indicus* [56].

Collectively, these results indicate that CHT may activate the plant responses through different signaling pathways involving different second messengers. In spite of this research, the mode of action of chitosan have not yet been elucidated clearly. This highlights the necessity for more studies in the future.

## 3. Application of CHT on Crop/Food Plants

The effect of CHT has been investigated in several crops, including cereal, ornamental, fruit, and medicinal plants.

### 3.1. Antipathogen Activities of CHT

The first study on antipathogen activity of CHT was published in 1979 by Allan and Hadwiger [57]. This study reported the fungicidal effect of CHT on fungi of different cell wall composition. This attracted the attention of agricultural industry on CHT since pathogens can cause severe diseases and relevant losses in crop yield and quality worldwide. Many pathogens can also produce toxins and metabolites, which can greatly affect the safety of agricultural products. Many studies reported the antimicrobial properties of CHT and its derivatives and many plant pathogens resulted sensitive to CHT [6]. Many studies deal with CHT effect on fungi. In fact, during both pre- and postharvest processes, infection of pathogenic fungi results in major losses of fruits and vegetables. At present, synthetic chemical fungicides are the primary choice to manage these pathogens. However, synthetic fungicides are potentially harmful on human health and their indiscriminate use induces the emergence of resistant strains. CHT is a promising alternative to control these diseases. In fact, it has been shown to possess a broad-spectrum fungicidal activity against several phytopathogenic fungi, effectively inhibiting their development at different life-cycle stages. For example, in pear (*Pyrus pyrifolia* L.) fruit CHT completely prevented germination and growth of *Alternaria kikuchiana* and *Physalospora piricola* [58]. CHT inhibited growth of *Botrytis cinerea* in liquid culture and suppressed grey mold disease caused by the fungus on detached grapevine leaves and bunch rot in Chardonnay and Sauvignon blanc wine grapes [59]. In rice (*Oryza sativa*) CHT showed marked antifungal activity against *Rhizoctonia solani*, the rice sheath blight pathogen [60]. Cowpea (*Vigna unguiculata* L.) plants were protected by CHT against *Fusarium oxysporum* infection [61]. CHT combined with clove oil or *Bacillus subtilis* endospores protected mature satsuma mandarin (*Citrus unshiu* Marc. cv. Miyagawawase) or Shogun mandarin oranges (*Citrus reticulate* Blanco cv. Shogun) fruits against *Penicillium digitatum*, the causal agent of citrus green mold [62,63]. Dragon fruit (*Hylocereus polyrhizus*) plants were protected by CHT against *Colletotrichum gloeosporioides* [64]. CHT enriched with lemongrass oil was very effective in in vivo and in vitro control of anthracnose caused by *Colletotrichum capsici* in bell pepper (*Capsicum annuum* L.) [65]. Finally, cell development and physiology of *Ustilago maydis*, the dimorphic fungus causing corn smut disease, were affected by CHT and glycol chitosan [66]. Scots pine (*Pinus sylvestris* L.) seedlings sprayed with CHT were effectively protected against parasitic damping-off and *Lophodermium* needle cast [67] and CHT induced cell death in spores of *Fusarium eumartii*, a fungal pathogen of potato and tomato [68]. CHT also prevented the growth of several pathogenic bacteria including *Xanthomonas* [69], *Pseudomonas syringae* [70], *Escherichia coli* [71], *Acidovorax citrulli* [72], *Agrobacterium tumefaciens*, and *Erwinia carotovora* [73]. However, CHT is generally less effective against bacteria than fungi and it often shows a higher effect on Gram-positive than Gram-negative bacteria, possibly due to the different composition of bacterial cell wall differently affecting the cellular entry of macromolecules such as CHT [74]. Compared with researches on antifungal and antibacterial action of CHT, its antiviral effect in plants is less documented. In bean plants, CHT induced resistance to the systemic pathogen bean mild mosaic virus [75]. In tobacco plants, CHT inhibited the appearance of localized necrotic lesions by systemic tobacco mosaic virus by 50%–90% [76] and the multiplication and movement of tobacco necrosis virus [32]. Recently, a protective action of CHT against other affecting plants pathogenic organisms has been also reported. Growth and larval vitality of oleander aphid *Aphis nerii* and cotton leafworm *Spodoptera littoralis* were severely affected by CHT [77]. CHT was very effective against the root-knot nematode *Meloidogyne javanica* in vitro and in vivo under greenhouse conditions [78]. The number of nymphs of the spear psylla, *Cacopsylla pyricola* (Foerster) (Hemiptera: Psyllidae), the key pest of cultivated pear (*Pyrus communis* L.), present on plants 30 days after releasing 10 adults on the tree was significantly reduced by foliar application of CHT [79]. Interestingly, the aphid predators ladybird (*Propylea japonica*, Coleoptera: Coccinellidae) fed with microcapsulae of Ca-alginate and CHT showed improved respiration and predation abilities compared to those fed with the liquid artificial diet usually utilized for mass rearing of predatory insects for biological control [80].

### 3.2. Stimulant Activity of CHT on Growth of Horticultural Plant

Recent studies on CHT use in horticultural plants are reported below. Foliar application of CHT enhanced fruit weight and productivity in tomato [81] and fruit yield, plant height, and leaf number in okra (*Hibiscus esculentus* L.) [82]. The application of CHT stimulated plant growth in Greek oregano [19] and increased biomass accumulation in cell cultures of three *Ocimum* species [83]. The soil addition of CHT increased height, canopy diameter, and leaf area of *Capsicum annuum* L. [84]. Seed dressing and foliar spraying at different growth stages with CHT impacted wheat (*Triticum aestivum* L.) production in large-scale field experiments by improving the yield components, such as the spike number and grains per spike [85]. CHT efficiently improved microspore embryogenesis and plantlet regeneration in *Brassica napus* L. [86]. Somatic embryogenesis in cultures of *Actinidia deliciosa* was enhanced by CHT [87]. CHT can also promote growth of flower species. CHT enhanced seed germination percentage of *Dendrobium formosum* orchid but was ineffective on *Dendrobium bigibbum* var. compactum [88]. Exposure of gladiolus corms (*Gladiolus communis* L.) to CHT accelerated the emergence and augmented the number of cormlets and flowers and prolonged their vase life [89]. Similarly, CHT application to freesia corms (*Freesia* Eckl. ex Klatt) led to taller plants, with more leaves, shoots, flowers, and corms [90]. The vase life of cut rose flowers (*Rosa hybrida* L.) “Red France” was also prolonged by CHT [91]. The largest number of studies on the effects of CHT deal with fruit crops. Foliar spraying with CHT enhanced coffee plant growth [92], and CHT spraying of strawberries (*Fragaria chiloensis* L.) stimulated vegetative growth and yield [93]. Dipping grapevine cuttings in CHT solution enhanced rooting and number of internodes [94]. Strawberry plants sprayed with CHT at different developmental stages produced fruits with increased shelf life [95]. The yield of tomato plants increased with CHT treatment [96].

CHT-based compounds can be also be used to coat or dip detached fruits, and this teatment prolongs shelf life and retards deterioration of many fruits such as cucumber, carrot, apple, citrus, kiwifruit, peach, pear, strawberry, and sweet cherry [97]. In fact, the protective barrier formed by CHT reduces water loss, inhibits gas exchange, decreases nutrient loss, and prevents microorganism growth on fruit surface [97]. Recent results have shown that CHT prolonged storage life and controlled decay of commercially important tropical fruits such as papaya and mango fruits [98]. CHT protected citrus (*Citrus limon* L.) fruit from mold disease caused by *Penicillium* spp. phytopathogenic fungi [99]. *Fragaria chiloensis* fruits treated with CHT and methyl jasmonate maintained higher levels of fruit firmness, anthocyanin, and showed significant delays in decay incidence compared to control fruit [95]. Shelf life of red kiwifruit (*Actinidia melanandra*) was extended by CHT coating [100]. Coating with CHT and trans-cinnamaldehyde improved structural integrity and antioxidant metabolism of fresh-cut Cantaloupe melons (*Cucumis melo* var. Cantalupensis Naud.) [101]. CHT coating improved quality and nutraceutical traits of loquat fruit (*Eriobotrya japonica* Lindl.) during postharvest life [102]. Postharvest CHT-*g*-salicylic acid application alleviated chilling injury and preserves cucumber (*Cucumis sativus* L.) fruit quality during cold storage through increased levels of salicylic acid and antioxidant enzymes [103].

## 4. Use of CHT Nanoparticles in Agriculture

The biggest goal of modern agriculture is to produce food of good quality and in sufficient quantity to meet the rise in world population limiting environmental impacts [104]. Agricultural production is severely affected by many pests and diseases that can cause considerable losses. During the past 100 years, chemical fertilizers and pesticides were used to face these problems and increase yield. The huge utilization of these products raised productivity significantly, but it also led to reduced biological diversity and degraded natural and agricultural systems. In addition, residue accumulation led to environmental pollution and public health problems, with development of resistant pests [105]. Therefore, alternative methods are necessary to face these problems of reducing the environmental impact of the activity without affecting agricultural productivity and with economic benefits for farmers. In this context, in recent years nanotechnology has emerged as a potential tool to greatly improve stress resistance and nutrients or chemicals uptake [106,107,108]. Nanotechnology-derived devices are currently employed as delivery systems to combat crop pathogens, to minimize nutrient losses during fertilization, and to improve yield [109]. The encapsulation of different chemicals in slow-release particles can be very valuable for sustainable agriculture and food safety [110]. Recently, CHT-based materials have been used to produce nanoparticles able to efficiently supply plants with chemicals and nutrients [111]. In fact, CHT easily absorbs to epidermis of leaves and stems prolonging the contact time and facilitating the uptake of the bioactive molecules.

### 4.1. Pesticide Delivery

In recent years, different studies describe the synthesis and utilization of CHT nanoparticles as a delivery system of pesticides. In addition, the encapsulation in CHT nanoparticles helps solve problems of solubility. For example, CHT microspheres were used to encapsulate synthetic brassinosteroids and diosgenin derivatives [112]. The water-insoluble botanical insecticide rotenone was encapsulated in nanomicelles composed of an amphiphilic derivative of CHT, increasing its solubility [113], and a material composed by carboxymethyl CHT and ricinoleic acid was synthesized and used to improve water solubility of the biopesticide azadirachtin [114]. These in vitro results encouraged in vivo studies. For example, microspheres composed of CHT and cashew tree gum were used to carry the essential oil of *Lippia sidoides*, an efficient insecticide against *A. aegypti* larvae [115] and microcapsules of CHT and sodium alginate were prepared and used for efficient imidacloprid release against the coleopteron *Martianus dermestoides* [116].

More recently, nanoparticles of CHT and sodium tripolyphosphate were prepared and successfully evaluated for suppression of rice blast fungus (*Pyricularia grisea*) [117] and oleoyl-chitosan nanoparticles were synthesized and used to disperse antifungal products [118].

### 4.2. Fertilizer and Micronutrient Delivery

It is well known that the availability of sufficient quantity of fertilizer and water as well as of microelements, i.e. Mn, Cu, B, Mo, Fe, Cl, and Zn, promotes optimal growth. Therefore, any improvement in plant yield requires a better utilization of growth nutrients and water resources. At present, it is estimated that the intended target plants cannot absorb about 80%–90% of phosphorus, 40%–70% of nitrogen, and 50%–70% of potassium contained in fertilizers. This resource loss is not only a waste of money but it also results in severe environmental pollution [105]. Farmers must move toward more efficient and sustainable practices to face the increase of fertilizer cost and the desire for environmentally-friendly agriculture. In this context, CHT-based polymers may be the solution [119]. Nanoparticles composed by an inner coating of CHT, an outer coating of crosslinked poly(acrylic acid)/diatomite—containing urea and a core of water-soluble granular nitrogen (N), phosphorus (P), and potassium (K) (NPK) fertilizer showed slow-controlled release of the nutrients without any detrimental impact on the soil [120]. A similar investigation was also performed by Corradini et al. [121], while Hussain et al. encapsulated urea in CHT microspheres obtaining a controlled release of the nutrient [122]. Recently, a potassium-containing, controlled-release biodegradable material based on CHT and montmorillonite clay layered silicate was prepared, and its structural, thermal, and morphological characterization was performed [123] and a new, super water-absorbing, material based on CHT, EDTA, and urea was synthesized [124]. This new material is very promising for its capacity to slow the release of urea and water as well as of metal ions that can be attached through the EDTA component.

It should be noted that the Green Revolution coupled with farming practices such as liming to increase crop productivity has progressively depleted soil level and availability of essential micronutrients such as boron, iron, copper, nickel, zinc, and molybdenum [125]. Up to now, micronutrients are commonly applied to leaves and nanoformulations of micronutrients may be sprayed on the leaves to promote absorption [126].

Alternatively, use of nanoparticles with micronutrients as a soil addition has been tested [119] and for their low toxicity those made from organic compounds like lipids, chitin, and CHT are more promising than the metal-made ones [127].

For an example, the delivery of microelements such as Zn^2+^ and Cu^2+^ was enhanced by cross-linked CHT/suberoyl chloride particles [128]. The same approach was used for the controlled delivery of other molecules important for plant growth. For example, CHT particles coupled with the phytohormone 1-naphthylacetic acid were synthesized and appeared able to slowly release the hormone [129]. These results are summarized in Table 2.

Over all, although preparation of nanoparticles is quite expensive, the lower use of nutrients to enhance plant productivity as well as the absence of undesirable environmental impact are compensatory benefits for the initial high cost.

### 4.3. Herbicide Delivery

In modern agriculture, the utilization of herbicides greatly increased to reduce the loss in food plants productivity and this excessive utilization caused severe environmental and human health problems [130], herbicides being the phytochemicals most widely contaminating hydrological systems. To manage these problems, the use of natural polysaccharides like CHT was investigated. For example, several authors synthesized and tested modified CHT nanoparticles to carry paraquat, the most widely used herbicide. Silva et al. demonstrated that alginate/CHT nanoparticles alter the release of the herbicide and its interaction with the soil [131]. In addition, CHT/tripolyphosphate nanoparticles decreased paraquat toxicity [132], and improved herbicidal activity against *Eichhornia crassipes* was observed when paraquat was encapsulated in silver/CHT nanoparticles [133]. Besides, an approach based on CHT nanoparticles may be used to monitor and remove excess of herbicide or heavy metals from soil and water. A nanocomposite material of CHT and montmorillonite was used to adsorb and remove the herbicide clopyralid present in water and soil [134]. Wen et al. demonstrated that CHT enhances the bioavailability of the chiral herbicide dichlorprop to the green alga *Chlorella pyrenoidosa* [135], and a plant esterase-CHT/gold nanoparticles−graphene nanosheet composite-based material was successfully tested for ultrasensitive detection of organophosphate pesticides in different samples [136]. CHT microcapsules from pollens of *Acer negundo*, *Cupressus sempervirens*, and *Populus nigra* were used to remove trace elements [137]. Similarly, spores of the phytopathogenic microfungi *Ustilago maydis* and *U. digitariae* were immobilized in cross-linked CHT matrix and used to remove trace elements from water [138].

### 4.4. Genetic Transformation

For efficient uptake of biological molecules such as DNA and plasmids into the plant cell, the biggest challenge is the cell wall. Traditional methods of delivery are expensive, relatively inefficient, and unsuccessful for genetic transformation of important crops such as monocotyledons [139]. Thus, the development of new methods based on nanotechnology is an important goal to obtain successful monocot and dicot transformants. In particular, the potential of use of CHT for genetic transformation is suggested by its capability to form, through electrostatic interactions, a complex where DNA is protected from nuclease degradation [6,140]. Interesting results were obtained by Wang et al. [141], who prepared QD-labeled CHT-DNA complexes to monitor nanoparticle-mediated genetic transformation of cultured cells of *Jatropha curcas*. This method gave rise to stable transformants with higher efficiency than other traditional methods of gene delivery.

Recent results showed that CHT-based nanoparticles with highly positive surface coatings can passively penetrate across the chloroplast membrane. Once in the chloroplast these nanoparticles exhibit both confined diffusion and convection, before reaching an irreversibly trapped state [142]. These results suggest that CHT nanoparticles can be used as possible molecular transporters into plastids like the chloroplast.

## 5. Conclusions

CHT induces numerous biological responses in plants such as stress resistance and increased productivity, which depend on CHT chemical composition and the timing and rate of application. Despite the huge amount of work, the mode of action of CHT is not yet completely unraveled and further transcriptomic and proteomic studies of defense genes and proteins are needed to fully understand the complex CHT-mediated responses, thus enabling a better use in plant disease control. Chitosan, as a unique abundant biopolymer, has a promising future in development of sustainable agricultural practices as well as in food production and preservation.

## Figures and Tables

**Table 1:** Proposed mechanisms for the antimicrobial effect of CHT.

Proposed CHT Action	Effect	Microorganism	References
Interaction with the phospholipids of microbial cell plasma membrane (CHT concentration <0.2 mg/mL)	Agglutination	gram-negative and gram-positive bacteria.	[33]
Interaction with the phospholipids of microbial cell plasma membrane	Disruption of bacterial cell membrane with leakage of intracellular substances	*E. coli*, *Staphylococcus aureus*	[34]
Interaction with proteins of microbial cell plasma membrane	Disruption of bacterial cell membrane integrity	*E. coli*, *Staphylococcus aureus*	[35]
Interaction with negatively charged components of the cell surface	Inhibition of H^+^-ATPase activity and chemiosmotic-driven transport	*Rhizopus stolonifer*	[36]
Interaction with microbial cell wall components	Disruption of cell wall integrity and alteration of intracellular ultrastructure	*Streptococcus sobrinus*, *Neisseria subflava*, *Candida albicans*	[37]
Chelation of metals	Inhibition of toxin production and microbial growth	*Alternaria alternata*	[38]
Interaction with the charged phosphate groups of DNA/RNA	Inhibition of the synthesis of mRNA and proteins	*E. coli*	[39]
Deposition on the bacterial surface (high m.w. CHT)	Blockage of nutrient flow	*E. coli*, *Bacillus cereus*	[40]

**Table 2:** Examples of fertilizers and micronutrients encapsulated in CHT-based controlled release matrix.

Matrices	Active Ingredient	Releasing Rate	References
CHT nanoparticles	NPK fertilizer	15% and 75% by the 3rd and 30th day, respectively.	[120]
CHT-methacrylic acid particles (diameter ca. 78 nm)	NPK fertilizer	n.d.	[121]
CHT microspheres (diameter ca. 200 mm)	urea	n.d.	[122]
CHT-montmorillonite microspheres (diameter ca. 200 mm)	KNO_3_	Fast for the first 3 days. Then continuum K release for at least 60 days.	[123]
CHT-EDTA	urea	n.d.	[124]
CHT-suberoyl chloride particles; crosslinking densities ranking from 0% to 7.4%	Zn^2+^; Cu^2+^	After 6 h ranking from 40 mg (0% density) to 15 mg (7.4% density).	[128]
CHT- phthalic anhydride	1-Naphthylacetic acid	Slow continuous release for several weeks. For example, at 20 °C, 10% and 25% by the 10th and 60th day, respectively.	[129]

## References

[1] Kurita K. (2006). Chitin and chitosan: Functional biopolymers from marine crustaceans. Mar. Biotech. (N.Y.).

[2] Park B.K., Kim M.M. (2010). Applications of chitin and its derivatives in biological medicine. Int. J. Mol. Sci..

[3] Hamed I., Ozogul F., Regenstein J.M. (2016). Industrial applications of crustacean by-products (chitin, chitosan, and chitooligosaccharides): A review. Trends Food Sci. Technol..

[4] Choi C., Nam J.P., Nah J.W. (2016). Application of chitosan and chitosan derivatives as biomaterials. J. Ind. Eng. Chem..

[5] Hamel L.-P., Beaudoin N. (2010). Chitooligosaccharide sensing and downstream signaling: Contrasted outcomes in pathogenic and beneficial plant-microbe interactions. Planta.

[6] Iriti M., Varoni E.M. (2015). Chitosan-induced antiviral activity and innate immunity in plants. Environ. Sci. Pollut. Res..

[7] Pichyangkuraa R., Chadchawanb S. (2015). Biostimulant activity of chitosan in horticulture. Sci. Horticul..

[8] Walker-Simmons M., Hadwiger L., Ryan C.A. (1983). Chitosans and pecticpolysaccharides both induce the accumulation of the antifungal phytoalexin pisatin in pea pods and antinutrient proteinase inhibitors in tomato leaves. Biochem. Biophys. Res. Commun..

[9] Ferri M., Tassoni A., Mackay R.G., Tait J.M. (2011). Chitosan as elicitor of health beneficial secondary metabolites in in vitro plant cell cultures. Handbook of Chitosan Research and Applications.

[10] Malerba M., Cerana R., Crosti P. (2011). Defense/stress responses activated by chitosan in sycamore cultured cells. Protoplasma.

[11] Cho M.H., No H.K., Prinyawiwatkul W. (2008). Chitosan treatments affect growth and selected quality of sunflower sprouts. J. Food Sci..

[12] Ali A., Muhammad M.T.M., Sijam K., Siddiqui Y. (2011). Effect of chitosan coatings on the physicochemical characteristics of Eksotika II papaya (*Carica papaya* L.) fruit during cold storage. Food Chem..

[13] Zhang D., Quantick P.C. (1997). Effects of chitosan coating on enzymatic browning and decay during postharvest storage of litchi (*Litchi chinensis* Sonn.) fruit. Postharv. Biol. Technol..

[14] Meng X., Tian S. (2009). Effects of preharvest application of antagonistic yeast combined with chitosan on decay and quality of harvested table grape fruit. J. Sci. Food Agric..

[15] Ghasemnezhad M., Shiri M.A., Sanavi M. (2010). Effect of chitosan coatings on some quality indices of apricot (*Prunus armeniaca* L.) during cold storage. Caspian J. Environ. Sci..

[16] Ghasemnezhad M., Nezhad M.A., Gerailoo S. (2011). Changes in postharvest quality of loquat (*Eriobotrya japonica*) fruits influenced by chitosan. Hortic. Environ. Biotechnol..

[17] Khan W.M., Prithiviraj B., Smith D.L. (2002). Effect of foliar application of chitin and chitosan oligosaccharides on photosynthesis of maize and soybean. Photosynthetica.

[18] Badawya M.E.I., Rabea E.I. (2009). Potential of the biopolymer chitosan with different molecular weights to control postharvest gray mold of tomato fruit. Postharvest Biol. Technol..

[19] Yin H., Frette X.C., Christensen L.P., Grevsen K. (2012). Chitosan oligosaccharides promote the content of polyphenols in Greek oregano (*Origanum vulgare* ssp. hirtum). J. Agric. Food Chem..

[20] Złotek U., Świeca M., Jakubczyk A. (2014). Effect of abiotic elicitation on main health-promoting compounds, antioxidant activity and commercial quality of butter lettuce (*Lactuca sativa* L.). Food Chem..

[21] Kim H., Chen F., Wang X., Rajapakse N.C. (2005). Effect of chitosan on the biological properties of sweet basil (*Ocimum basilicum* L.). J. Agric. Food Chem..

[22] Singh S. (2016). Enhancing phytochemical levels, enzymatic and antioxidant activity of spinach leaves by chitosan treatment and an insight into the metabolic pathway using DART-MS technique. Food Chem..

[23] Kohle H., Jeblick W., Poten F., Blaschek W., Kauss H. (1985). Chitosan-elicited callose synthesis in soybean cells as a Ca^2+^-dependent process. Plant Physiol..

[24] Faoro F., Iriti M. (2007). Callose synthesis as a tool to screen chitosan efficacy in inducing plant resistance to pathogens. Caryologia.

[25] Zuppini A., Baldan B., Millioni R., Favaron F., Navazio L., Mariani P. (2003). Chitosan induces Ca^2+^ mediated programmed cell death in soybean cells. New Phytol..

[26] Yin H., Zhao X., Bai X., Du Y. (2010). Molecular cloning and characterization of a *Brasica napus* L. MAP kinase involved in oligochitosan-induced defense signaling. Plant Mol. Biol. Rep..

[27] Amborabé B.E., Bonmort J., Fleurat-Lessard P., Roblin G. (2008). Early events induced by chitosan on plant cells. J. Exp. Bot..

[28] Hadwiger L.A. (2008). Pea-*Fusarium solani* interactions contributions of a system toward understanding disease resistance. Phytopathology.

[29] Iriti M., Faoro F. (2009). Chitosan as a MAMP searching for a PRR. Plant Signal. Behav..

[30] Orlita A., Sidwa-Gorycka M., Paszkiewicz M., Malinski E., Kumirska J., Siedlecka E.M. (2008). Application of chitin and chitosan as elicitors of coumarins and fluoroquinolone alkaloids in *Ruta graveolens* L. (common rue). Biotech. Appl. Biochem..

[31] Doares S.H., Syrovets T., Weiler E.W., Ryan C.A. (1995). Oligogalacturonides and chitosan activate plant defence genes through the octadecanoic pathway. Proc. Natl. Acad. Sci. USA.

[32] Iriti M., Faoro F. (2008). Abscisic acid mediates the chitosan-induced resistance to tobacco necrosis virus (TNV). Plant Physiol. Biochem..

[33] Sudarshan N.R., Hoover D.G., Knorr D. (1992). Antibacterial action of chitosan. Food Biotechnol..

[34] Liang C., Yuan F., Liu F., Wang Y., Gao Y. (2014). Structure and antimicrobial mechanism of ε-polylysine-chitosan conjugates through Maillard reaction. Int. J. Biol. Macromol.,.

[35] Xing K., Chen X.G., Liu C.S., Cha D.S., Park H.J. (2009). Oleoyl-chitosan nanoparticles inhibits *Escherichia coli* and *Staphylococcus aureus* by damaging the cell membrane and putative binding to extracellular or intracellular targets. Int. J. Food Microbiol..

[36] García-Rincóna J., Vega-Pérez J., Guerra-Sánchez M.G., Hernández-Lauzardo A.N., Peña-Díaz A., Velázquez-Del Valle M.G. (2010). Effect of chitosan on growth and plasma membrane properties of *Rhizopus stolonifer* (Ehrenb.:Fr.) Vuill. Pestic. Biochem. Phys..

[37] Geisberger G., Gyenge E.B., Hinger D., Käch A., Maake C., Patzke G.R. (2013). Chitosan-thioglycolic acid as a versatile antimicrobial agent. Biomacromolecules.

[38] Reddy M.V.B., Arul J., Ait-Barka E., Angers P., Richard C., Castaigne F. (1998). Effect of chitosan on growth and toxin production by *Alternaria alternata* f. sp. lycopersici. Biocontrol. Sci. Technol..

[39] Liu X.F., Guan Y.L., Yang D.Z., Li Z., Yao K.D. (2001). Antibacterial action of chitosan and carboxymethylated chitosan. J. Appl. Polym. Sci..

[40] Vishu K.A.B., Varadaraj M.C., Gowda L.R., Tharanathan R.N. (2005). Characterization of chito-oligosaccharides prepared by chitosanolysis with the aid of papain and Pronase, and their bactericidal action against *Bacillus cereus* and *Escherichia coli*. Biochem. J..

[41] Rabea E.I., Badawy M.E.T., Stevens C.V., Smagghe G., Steurbaut W. (2003). Chitosan as antimicrobial agent: Applications and mode of action. Biomacromolecules.

[42] Hadwiger L.A. (2015). Anatomy of a nonhost disease resistance response of pea to *Fusarium solani*: PR gene elicitation via DNase, chitosan and chromatin alterations. Front. Plant Sci..

[43] Chen H.P., Xu L.L. (2005). Isolation and characterization of a novel chitosan-binding protein from non-heading Chinese cabbage leaves. J. Integr. Plant Biol..

[44] Petutschnig E.K., Jones A.M.E., Serazetdinova L., Lipka U., Lipka V. (2010). The Lysin Motif Receptor-like Kinase (LysM-RLK) CERK1 is a major chitin-binding protein in *Arabidopsis thaliana* and subject to chitin-induced phosphorylation. J. Biol. Chem..

[45] Kaku H., Nishizawa Y., Ishii-Minami N., Akimoto-Tomiyama C., Dohmae N., Takio K., Minami E., Shibuya N. (2006). Plant cells recognize chitin fragments for defence signalling through a plasma membrane receptor. Proc. Natl. Acad. Sci. USA.

[46] Povero G., Loreti E., Pucciariello C., Santaniello A., Di Tommaso D., Di Tommaso G., Kapetis D., Zolezzi F., Piaggesi A., Perata P. (2011). Transcript profiling of chitosan-treated Arabidopsis seedings. J. Plant Res..

[47] Faoro F., Maffi D., Cantù D., Iriti M. (2008). Chemical-induced resistance against powdery mildew in barley: The effects of chitosan and benzothiadiazole. BioControl.

[48] Iriti M., Sironi M., Gomarasca S., Casazza A.P., Soave C., Faoro F. (2006). Cell death-mediated antiviral effect of chitosan in tobacco. Plant Physiol. Biochem..

[49] Hadwiger L.A. (2013). Multiple effects of chitosan on plant systems: Solid science or hype. Plant Sci..

[50] Raho N., Ramirez L., Lanteri M.L., Gonorazky G., Lamattina L., ten Have A., Laxalt A.M. (2011). Phosphatidic acid production in chitosan-elicited tomato cells, via both phospholipase D and phospholipase C/diacylglycerol kinase, requires nitric oxide. J. Plant Physiol..

[51] Zhang H., Zhao X., Yang J., Yin H., Wang W., Lu H., Du Y. (2011). Nitric oxide production and its functional link with OIPK in tobacco defense response elicited by chitooligosaccharide. Plant Cell Rep..

[52] Malerba M., Cerana R. (2015). Reactive oxygen and nitrogen species in defense/stress responses activated by chitosan in sycamore cultured cells. Int. J. Mol. Sci..

[53] Rakwal R., Tamogami S., Agrawal G.K., Iwahashi H. (2002). Octadecanoid signaling component “burst” in rice (*Oryza sativa* L.) seedling leaves upon wounding by cut and treatment with fungal elicitor chitosan. Biochem. Biophys. Res. Commun..

[54] Yin H., Li S., Zhao X., Du Y., Ma X. (2006). cDNA microarray analysis of gene expression in *Brassica napus* treated with oligochitosan elicitor. Plant Physiol. Biochem..

[55] Flors V., Ton J., Jakab G., Mauch-Mani B. (2005). Abscisic acid and callose: Team players in defence against pathogens?. J. Phytopathol..

[56] Safaei Z., Karimi K., Golkar P., Zamani A. (2015). Effects of plant growth hormones on *Mucor indicus* growth and chitosan and ethanol production. Int. J. Mol. Sci..

[57] Allan C.R., Hadwiger L.A. (1979). The fungicidal effect of chitosan on fungi of varying cell composition. Exp. Mycol..

[58] Meng X.H., Yang L.Y., Kennedy J.F., Tian S.P. (2010). Effects of chitosan and oligochitosan on growth of two fungal pathogens and physiological properties in pear fruit. Carbohydr. Polym..

[59] Reglinski T., Elmer P.A.G., Taylor J.T., Wood P.N., Hoyte S.M. (2010). Inhibition of *Botrytis cinerea* growth and suppression of botrytis bunch rot in grapes using chitosan. Plant Pathol..

[60] Liu H., Tian W.X., Li B., Wu G.X., Ibrahim M., Tao Z.Y., Wang Y.L., Xie G.L., Li H.Y., Sun G.C. (2012). Antifungal effect and mechanism of chitosan against the rice sheath blight pathogen, *Rhizoctonia solani*. Biotechnol. Lett..

[61] Berger L.R.R., Stamford N.P., Willadino L.G., Laranjeira D., de Lima M.A.B., Malheiros S.M.M., de Oliveira W.J., Stamford T.C.M. (2016). Cowpea resistance induced against *Fusarium oxysporum* f. sp. tracheiphilum by crustaceous chitosan and by biomass and chitosan obtained from *Cunninghamella elegans*. Biol. Control.

[62] Shao X., Cao B., Xu F., Xie S., Yu D., Wang H. (2015). Effect of postharvest application of chitosan combined with clove oil against citrus green mold. Postharvest Biol. Technol..

[63] Waewthongraka W., Pisuchpen S., Leelasuphakul W. (2015). Effect of *Bacillus subtilis* and chitosan applications on green mold (*Penicilium digitatum* Sacc.) decay in citrus fruit. Postharvest Biol. Technol..

[64] Zahid N., Maqbool M., Siddiqui Y., Manickam S., Ali A. (2015). Regulation of inducible enzymes and suppression of anthracnose using submicron chitosan dispersions. Sci. Hortic..

[65] Ali A., Noh N.M., Mustafa M.A. (2015). Antimicrobial activity of chitosan enriched with lemongrass oil against anthracnose of bell pepper. Food Pack. Shel. Life.

[66] Olicón-Hernández D.R., Hernández-Lauzardo A.N., Pardo J.P., Peña A., Velázquez-del Valle M.G., Guerra-Sánchez G. (2015). Influence of chitosan and its derivatives on cell development and physiology of *Ustilago maydis*. Int. J. Biol. Macromol..

[67] Aleksandrowicz-Trzcińska M., Bogusiewicz A., Szkop M., Drozdowski S. (2015). Effect of chitosan on disease control and growth of Scots Pine (*Pinus sylvestris* L.) in a forest nursery. Forests.

[68] Terrile M.C., Mansilla A.Y., Albertengo L., Rodríguez M.S., Anahí Casalongué C. (2015). Nitric-oxide-mediated cell death is triggered by chitosan in *Fusarium eumartii* spores. Pest Manag. Sci..

[69] Li B., Wang X., Chen R.X., Huangfu W.G., Xie G.L. (2008). Antibacterial activity of chitosan solution against *Xanthomonas* pathogenic bacteria isolated from *Euphorbia pulcherrima*. Carbohyd. Polym..

[70] Mansilla A.Y., Albertengo L., Rodríguez M.S., Debbaudt A., Zúñiga A., Casalongué C.A. (2013). Evidence on antimicrobial properties and mode of action of a chitosan obtained from crustacean exoskeletons on *Pseudomonas syringae* pv. tomato DC3000. Appl. Microbiol. Biotechnol..

[71] Goñi M.G., Tomadoni B., Roura S.I., Moreira M.R. (2014). Effect of preharvest application of chitosan and tea tree essential oil on postharvest evolution of lettuce native microflora and exogenous *Escherichia coli* O157:H7. J. Food Safe.

[72] Li B., Shi Y., Shan C.L., Zhou Q., Ibrahim M., Wang Y.L., Wu G.X., Li H.Y., Xie G.L., Sun G.C. (2013). Effect of chitosan solution on the inhibition of *Acidovorax citrulli* causing bacterial fruit blotch of watermelon. J. Sci. Food Agric..

[73] Badawy M.E., Rabea E.I., Taktak N.E. (2014). Antimicrobial and inhibitory enzyme activity of *N*-(benzyl) and quaternary *N*-(benzyl) chitosan derivatives on plant pathogens. Carbohydr. Polym..

[74] Xing K., Zhu X., Peng X., Qin S. (2015). Chitosan antimicrobial and eliciting properties for pest control in agriculture: A review. Agron. Sustain. Dev..

[75] Kulikov S.N., Chirkov S.N., Il’ina A.V., Lopatin S.A., Varlamov V.P. (2006). Effect of molecular weight of chitosan on its antiviral activity in plants. Appl. Biochem. Microbiol..

[76] Davydova V.N., Nagorskaya V.P., Gorbach V.I., Kalitnik A.A., Reunov A.V., Solov’eva T.F., Ermak I.M. (2011). Chitosan antiviral activity: Dependence on structure and depolymerization method. Appl. Biochem. Microb..

[77] Badawy M.E.I., Ahmed F. (2012). El-Aswad, A.F. Insecticidal activity of chitosans of different molecular weights and chitosan-metal complexes against cotton leafworm *Spodoptera littoralis* and oleander aphid *Aphis nerii*. Plant Protect. Sci..

[78] El-Sayed S.M., Mahdy M.E. (2015). Effect of chitosan on root-knot nematode, *Meloidogyne javanica* on tomato plants. Int. J. ChemTech. Res..

[79] Cooper W.R., Horton D.R. (2015). Effects of elicitors of host plant defenses on pear psylla, *Cacopsylla pyricola*. Entomol. Exp. Appl..

[80] Tan X.L., Zhao J., Wang S., Zang F. (2015). Optimization and evaluation of microencapsulated artificial diet for mass rearing the predatory ladybird *Propylea japonica* (Coleoptera: Coccinellidae). Insect Sci..

[81] Sathiyabama M., Akila G., Einstein Charles R. (2014). Chitosan-induced defence responses in tomato plants against early blight disease caused by *Alternaria solani* (Ellis and Martin) Sorauer. Arch. Phytopathol. Plant Prot..

[82] Mondal M.M.A., Malek M.A., Puteh A.B., Ismail M.R., Ashrafuzzaman M., Naher L. (2012). Effect of foliar application of chitosan on growth and yield in okra. Aust. J. Crop Sci..

[83] Mathew R., Sankar P.D. (2012). Effect of methyl jasmonate and chitosan on growth characteristics of *Ocimum basilicum* L., *Ocimum sanctum* L. and *Ocimum gratissimum* L. cell suspension cultures. Afr. J. Biotechnol..

[84] Chookhongkha N., Miyagawa S., Jirakiattikul Y., Photchanachai S. Chili growth and seed productivity as affected by chitosan. Proceedings of the International Conference on Agriculture Technology and Food Sciences (ICATFS’2012).

[85] Wang M., Chen Y., Zhang R., Wang W., Zhao X., Du Y., Yin H. (2015). Effects of chitosan oligosaccharides on the yield components and production quality of different wheat cultivars (*Triticum aestivum* L.) in Northwest China. Field Crops Res..

[86] Ahmadi B., Shariatpanahi M.E. (2015). Proline and chitosan enhanced efficiency of microspore embryogenesis induction and plantlet regeneration in *Brassica napus* L. Plant Cell Tiss. Org. Cult..

[87] Corsi B., Riccioni L., Forni C. (2015). In vitro cultures of *Actinidia deliciosa* (A. Chev) C.F. Liang & A.R. Ferguson: A tool to study the SAR induction of chitosan treatment. Org. Agric..

[88] Kananont N., Pichyangkura R., Chanprame S., Chadchawan S., Limpanavech P. (2010). Chitosan specificity for the in vitro seed germination of two *Dendrobium* orchids (Asparagales: Orchidaceae). Sci. Hortic..

[89] Ramos-García M., Ortega-Centeno S., Hernández-Lauzardo A.N., Alia-Tejacal I., Bosquez-Molina E., Bautista-Baños S. (2009). Response of gladiolus (*Gladiolus* spp.) plants after exposure corms to chitosan and hot water treatments. Sci. Hortic..

[90] Salachna P., Zawadzińska A. (2014). Effect of chitosan on plant growth, flowering and corms yield of potted freesia. J. Ecol. Eng..

[91] Jing H.J., Li H.Q. (2015). Chitooligosaccharide prolongs vase life of cut roses by decreasing reactive oxygen species. Kor. J. Hort. Sci. Technol..

[92] Van S.N., Minh H.D., Anh D.N. (2013). Study on chitosan nanoparticles on biophysical characteristics and growth of Robusta coffee in green house. Biocatal. Agric. Biotechnol..

[93] El-Miniawy S., Ragab M., Youssef S., Metwally A. (2013). Response of strawberry plants to foliar spraying of chitosan. Res. J. Agric. Biol. Sci..

[94] Górnik K., Grzesik M., Romanowska-Duda B. (2008). The effect of chitosan on rooting of grapevine cuttings and on subsequent plant growth under drought and temperature stress. J. Fruit Ornam. Plant Res..

[95] Saavedra G.M., Figueroa N.E., Poblete L.A., Cherian S., Figueroa C.R. (2016). Effects of preharvest applications of methyl jasmonate and chitosan on postharvest decay, quality and chemical attributes of *Fragaria chiloensis* fruit. Food Chem..

[96] Sathiyabama M., Charles R.E. (2015). Fungal cell wall polymer based nanoparticles in protection of tomato plants from wilt disease caused by *Fusarium oxysporum* f.sp. lycopersici. Carbohydr. Polym..

[97] Kerch G. (2015). Chitosan films and coatings prevent losses of fresh fruit nutritional quality: A review. Trends Food Sci. Technol..

[98] Hewajulige I.G.N., Wilson Wijeratnam R.S., Perera M.G.D.S., Fernando S.A. (2015). Extending storage life of commercially important tropical fruits using bio-waxes. Acta Horticul..

[99] Tayel A.A., Moussa S.H., Salem M.F., Mazrou K.E., El-Tras W.F. (2016). Control of citrus molds using bioactive coatings incorporated with fungal chitosan/plant extracts composite. J. Sci. Food Agric..

[100] Kayaa M., Cesoniene L., Daubaras R., Leskauskaite D., Zabulione D. (2016). Chitosan coating of red kiwifruit (*Actinidia melanandra*) for extending of the shelf life. Int. J. Biol. Macromol..

[101] Carvalho R.L., Cabral M.F., Germano T.A., de Carvalho W.M., Brasil I.M., Gallão M.I., Moura C.F.H., Lopes M.M.A., de Miranda M.R.A. (2016). Chitosan coating with trans-cinnamaldehyde improves structural integrity and antioxidant metabolism of fresh-cut melon. Postharv. Biol. Technol..

[102] Petriccione M., Pasquariello M.S., Mastrobuoni F., Zampella L., Di Patre D., Scortichini M. (2015). Influence of a chitosan coating on the quality and nutraceutical traits of loquat fruit during postharvest life. Sci. Hortic..

[103] Zhang Y., Zhang M., Yang H. (2015). Postharvest chitosan-*g*-salicylic acid application alleviates chilling injury and preserves cucumber fruit quality during cold storage. Food Chem..

[104] FAO Statistical Yearbook 2013. http://www.fao.org/docrep/018/i3107e/i3107e00.htm).

[105] Sun B., Zhang L., Yang L., Zhang F., Norse D., Zhu Z. (2012). Agricultural non-point source pollution in China: Causes and mitigation measures. Ambio.

[106] Ghormade V., Deshpande M.V., Paknikar K.M. (2011). Perspectives for nano-biotechnology enabled protection and nutrition of plants. Biotechnol. Adv..

[107] Khot L.R., Sankaran S., Maja J.M., Ehsani R., Schuster E.W. (2012). Applications of nanomaterials in agricultural production and crop protection: A review. Crop Prot..

[108] Kah M., Hofmann T. (2014). Nanopesticide research: Current trends and future priorities. Environ. Int..

[109] Handford C.E., Dean M., Henchion M., Spence M., Elliott C.T., Campbell K. (2014). Implications of nanotechnology for the agri-food industry: Opportunities, benefits and risks. Trends Food Sci. Technol..

[110] Cota-Arriola O., Cortez-Rocha M.O., Burgos-Hernández A., Ezquerra-Brauer J.M., Plascencia-Jatomea M. (2013). Controlled release matrices and micro/nanoparticles of chitosan with antimicrobial potential: Development of new strategies for microbial control in agriculture. J. Sci. Food Agric..

[111] Kah M., Tiede K., Beulke S., Hofmann T. (2013). Nanopesticides: State of knowledge, environmental fate, and exposure modeling. Crit. Rev. Environ. Sci. Technol..

[112] Quiñones J.P., García Y.C., Curiel H., Covas C.P. (2010). Microspheres of chitosan for controlled delivery of brassinosteroids with biological activity as agrochemicals. Carbohydr. Polym..

[113] Lao S.B., Zhang Z.X., Xu H.H., Jiang G.B. (2010). Novel amphiphilic chitosan derivatives: Synthesis, characterization and micellar solubilization of rotenone. Carbohydr. Polym..

[114] Feng B.H., Peng L.F. (2012). Synthesis and characterization of carboxymethyl chitosan carrying ricinoleic functions as an emulsifier for azadirachtin. Carbohydr. Polym..

[115] Paula H.C.B., Sombra F.M., Cavalcante R.F., Abreu F.O.M.S., de Paula R.C.M. (2011). Preparation and characterization of chitosan/cashew gum beads loaded with *Lippia sidoides* essential oil. Mater. Sci. Eng. C.

[116] Guan H., Chi D., Yu J., Li X. (2008). A novel photodegradable insecticide: Preparation, characterization and properties evaluation of nano-Imidacloprid. Pest. Biochem. Physiol..

[117] Manikandan A., Sathiyabama M. (2016). Preparation of chitosan nanoparticles and its effect on detached rice leaves infected with *Pyricularia grisea*. Int. J. Biol. Macromol..

[118] Xing K., Shen X., Zhu X., Ju X., Miao X., Tian J., Feng Z., Peng X., Jiang J., Qin S. (2016). Synthesis and in vitro antifungal efficacy of oleoyl-chitosan nanoparticles against plant pathogenic fungi. Int. J. Biol. Macromol..

[119] Davidson D., Gu F.X. (2012). Materials for sustained and controlled release of nutrients and molecules to support plant growth. J. Agric. Food Chem..

[120] Wu L., Liu M., Liang R. (2008). Preparation and properties of a double-coated slow-release NPK compound fertilizer with superabsorbent and water-retention. Bioresour. Technol..

[121] Corradini E., de Moura M.R., Mattoso L.H.C. (2010). A preliminary study of the incorparation of NPK fertilizer into chitosan nanoparticles. Express Polym. Lett..

[122] Hussain M.R., Devi R.R., Maji T.K. (2012). Controlled release of urea from chitosan microspheres prepared by emulsification and cross-linking method. Iran. Polym. J..

[123] Santos B.R.D., Bacalhau F.B., Pereira T.D.S., Souza C.F., Faez R. (2015). Chitosan-Montmorillonite microspheres: A sustainable fertilizer delivery system. Carbohydr. Polym..

[124] Narayanan A., Dhamodharan R. (2015). Super water-absorbing new material from chitosan, EDTA and urea. Carbohydr. Polym..

[125] Alloway B., Alloway B.J. (2008). Micronutrient Deficiencies in Global Crop Production.

[126] Martens D.C., Westermann D.T. (1991). Micronutrients in Agriculture.

[127] Peteu S.F., Oancea F., Sicuia O.A., Constantinescu F., Dinu S. (2010). Responsive polymers for crop protection. Polymers.

[128] Chen C., Gao Z., Qiu X., Hu S. (2013). Enhancement of the controlled-release properties of chitosan membranes by crosslinking with suberoyl chloride. Molecules.

[129] Tao S., Pang R., Chen C., Ren X., Hu S. (2012). Synthesis, characterization and slow release properties of *O*-naphthylacetyl chitosan. Carbohydr. Polym..

[130] Pundir C.S., Chauhan N. (2012). Acetylcholinesterase inhibition-based biosensors for pesticide determination: A review. Anal. Biochem..

[131] Silva M.S., Cocenza D.S., Grillo R., de Melo N.F.S., Tonello P.O.S., deOliveira L.C., Cassimiro D.L., Rosa A.H., Fraceto L.F. (2011). Paraquat-loaded alginate/chitosan nanoparticles: Preparation, characterization and soil sorption studies. J. Hazard. Mater..

[132] Grillo R., Pereira A.E.S., Nishisaka C.S., de Lima R., Oehlke K., Greiner R., Fraceto L.F. (2014). Chitosan/tripolyphosphate nanoparticles loaded with paraquat herbicide: An environmentally safer alternative for weed control. J. Hazard. Mater..

[133] Namasivayam S.K.R., Aruna A., Gokila G. (2014). Evaluation of silver nanoparticles-chitosan encapsulated synthetic herbicide paraquat (AgNp-CS-PQ) preparation for the controlled release and improved herbicidal activity against *Eichhornia crassipes*. Res. J. Biotechnol..

[134] Celis R., Adelino M.A., Hermosín M.C., Cornejo J. (2012). Montmorillonite–chitosan bionanocomposites as adsorbents of the herbicide clopyralid in aqueous solution and soil/water suspensions. J. Hazard. Mater..

[135] Wen Y.Z., Chen H., Yuan Y.L., Xu D.M., Kang X.D. (2011). Enantioselective ecotoxicity of the herbicide dichlorprop and complexes formed with chitosan in two fresh water green algae. J. Environ. Monit..

[136] Bao J., Hou C., Chen M., Li J., Huo D., Yang M., Luo X., Lei Y. (2015). Plant esterase–chitosan/gold nanoparticles–graphene nanosheet composite-based biosensor for the ultrasensitive detection of organophosphate pesticides. J. Agric. Food Chem..

[137] Sargın I., Kaya M., Arslan G., Baran T., Ceter T. (2015). Preparation and characterisation of biodegradable pollen–chitosan microcapsules and its application in heavy metal removal. Biores.Technol..

[138] Sargın I., Arslan G., Kaya M. (2016). Microfungal spores (*Ustilago maydis* and *U. digitariae*) immobilized chitosan microcapsules for heavy metal removal. Carbohydr. Polym..

[139] Krenek P., Samajova O., Luptovciak I., Doskocilova A., Komis G., Samaj J. (2015). Transient plant transformation mediated by *Agrobacterium tumefaciens*: Principles, methods and applications. Biothecnol. Adv..

[140] Mao S., Sun W., Kissel T. (2010). Chitosan-based formulations for delivery of DNA and siRNA. Adv. Drug Deliv. Rev..

[141] Wang Q., Chen J.N., Zhan P., Zhang L., Kong Q.Q. (2013). Establishment of a suspension cell system for transformation of *Jatropha curcas* using nanoparticles. Adv. Mater. Res..

[142] Wong M.H., Misra R.P., Giraldo J.P., Kwak S.Y., Son Y., Landry M.P., Swan J.W., Blankschtein D., Strano M.S. (2016). Lipid exchange envelope penetration (LEEP) of nanoparticles for plant engineering: A universal localization mechanism. Nano Lett..

